# Contemporary short-term outcomes of surgery for aortic stenosis: transcatheter vs. surgical aortic valve replacement

**DOI:** 10.1007/s11748-021-01672-8

**Published:** 2021-06-22

**Authors:** Shunsuke Saito, Toshimi Sairenchi, Masahiro Tezuka, Yusuke Takei, Go Tsuchiya, Koji Ogata, Osamu Monta, Ikuko Shibasaki, Yasushi Tsutsumi, Hirotsugu Fukuda

**Affiliations:** 1 Department of Cardiac and Vascular Surgery, Dokkyo Medical University, 880 Kitakobayashi, Shimotsugagun, Mibu, Tochigi 321-0293 Japan; 2 Center for Research Collaboration and Support, Comprehensive Research Facilities for Advanced Medical Science, Dokkyo Medical University, Mibu, Japan; 3 Department of Cardiovascular Surgery, Fukui Cardiovascular Center, Fukui, Japan

**Keywords:** Aortic stenosis, Transcatheter aortic valve replacement, Surgical aortic valve replacement

## Abstract

**Objectives:**

This study aimed to compare the short-term outcomes of transcatheter and surgical aortic valve replacements (TAVR and SAVR) in high-, intermediate-, and low-preoperative risk patients.

**Methods:**

A total of 454 patients who underwent TAVR or SAVR were included. Patients were categorized into high-, intermediate-, and low-risk according to the Society of Thoracic Surgery-Predicted Risk of Mortality score and clinical outcomes were compared between TAVR and SAVR groups.

**Results:**

TAVR was less invasive, with less bleeding and transfusion (*p* < 0.001), less frequent new-onset atrial fibrillation (*p* < 0.001), and shorter intensive care unit stay (*p* < 0.001). Furthermore, transcatheter valves performed better than surgical valves, with lower peak velocity (*p* = 0.003) and pressure gradient (*p* < 0.001) and higher effective orifice area index (*p* < 0.001). The clinical outcomes of TAVR were comparable to or even superior to those of SAVR in high- and intermediate-risk patients. In low-risk patients, the 1- and 2-year mortality rates were 6.3% and 12.1%, respectively, in the TAVR group and 0% and 0.9%, respectively, in the SAVR group (*p* < 0.001). Mild or greater paravalvular leakage was a risk factor for mortality (hazard ratio 35.78; *p* < 0.001).

**Conclusions:**

TAVR was superior to SAVR in the sense of less invasiveness and valvular function. However, the indication of TAVR in low-risk patients should be carefully discussed, because paravalvular leakage was a risk factor for short-term mortality.

**Supplementary Information:**

The online version contains supplementary material available at 10.1007/s11748-021-01672-8.

## Introduction

Surgical aortic valve replacement (SAVR) has long been the gold standard for the surgical treatment of severe aortic stenosis (AS). Over the last decade, transcatheter aortic valve replacement (TAVR) has revolutionized the treatment of severe AS. Early studies showed a clear benefit of TAVR in prohibitive and high-surgical-risk patients (Society of Thoracic Surgery-Predicted Risk of Mortality [STS-PROM] > 8%), [1–3] and intermediate-risk patients (STS-PROM 4–8%) [4, 5]. In addition, two comparative trials among low-risk patients also reported promising results [6, 7]. Most guidelines derive their current indications from these industry-driven randomized controlled trials (RCTs) [8, 9].

The results of RCTs have often been criticized because of the limited comparability to “real-world” practice. The surgical community has further questioned these because of the potential conflicts of interest and the perceived “suboptimal” outcomes for SAVR [10–13]. However, before criticizing the RCT outcome, it is essential for us to learn the accurate outcomes of our “real-world” practice in our own country, but real-world data comparing TAVR and SAVR in Japan are scarce [14]. As such, the purpose of the present study was to investigate the preoperative status and clinical outcomes of patients who underwent surgical treatment for severe AS in recent years and evaluate whether we could apply the RCT results in our daily practice.

## Methods

### Patients

Ethics approval was obtained from the appropriate ethics committee for this retrospective study. From October 2015 to July 2019, 678 patients underwent aortic valve replacement (AVR) at our institutes. Of the 678 patients, 224 underwent AVR concomitantly with aortic or mitral surgery, left ventricular restoration, left ventricular assist device implantation, or AVR for aortic insufficiency or infective endocarditis and were excluded from this study (Supplemental Fig. 1). The remaining 454 patients who underwent AVR for severe AS were then included in this study. Patients were divided into the TAVR and SAVR groups, and clinical outcomes were compared. For the mortality and morbidity analyses, patients were categorized into 3 groups according to preoperative STS-PROM score: STS-PROM ≥ 8.0 (high-risk group), 4.0 ≤ STS-PROM < 8.0 (intermediate-risk group), and STS-PROM < 4.0 (low-risk group) (Supplemental Fig. 1). Mortality and morbidity were compared between the TAVR and SAVR groups.

### Selection of treatment

The selection of treatment (TAVR or SAVR) has always been performed through discussions within the heart team, consisted of cardiologists, surgeons, anesthetists, nurses, and clinical engineers. Transcatheter approach or open surgical approach was chosen considering the patient’s age, severity of condition, frailty, cognitive function, and comorbidities. All the procedures were done under general anesthesia and with the use of transesophageal echocardiography. The approach in TAVR group (transfemoral, transapical, or other alternative approaches) were chosen through discussions within the heart team considering the anatomical characteristics of the patient. SAVR was done through median sternotomy and under cardiac arrest with cardiopulmonary bypass support.

### Data collection

Data were extracted from patient charts recorded in the hospital’s computer database. Follow-up started on the day of TAVR or SAVR and was censored at 30 months. Postoperative echocardiographic study was done one week after the procedure in most of the patients. In some patients in TAVR group, echocardiography was also done 1 day after the procedure to rule out complications such as cardiac tamponade. The first postoperative echocardiographic data was used for the analysis.

### Statistical analysis

Continuous variables are presented as mean ± SD, and categorical variables are presented as numbers and proportions. All continuous variables were checked for normal distribution using the Shapiro–Wilk test and a normal probability plot. For univariate analyses, normally distributed variables were compared using Student’s *t* test, non-normally distributed variables were compared using the Mann–Whitney *U* test, categorical variables were compared using Chi-squared analysis or Fisher’s exact test, as appropriate. Time-to-event analyses were performed using Kaplan–Meier estimates and compared with the use of the log-rank test. The predictors of mortality were evaluated using the Cox hazard model. Factors with a value of *p* < 0.10 in the univariate analysis were included in the multivariable analysis.

In the low-risk group, the comparison between TAVR and SAVR was further done using propensity score matching. From the data of 66 TAVR patients and 121 SAVR patients, a multivariable logistic regression model was used to develop propensity score for the selection of TAVR with seven variables from baseline characteristics that were significantly different between TAVR and SAVR groups. The c statistics was 0.937.

All two-sided *p* values less than 0.05 were considered to be statistically significant.

All statistical analyses were performed using EZR (Saitama Medical Center, Jichi Medical University, Saitama, Japan), a graphical user interface for R (The R Foundation for Statistical Computing, Vienna, Austria). More precisely, it is a modified version of R commander designed to add statistical functions frequently used in biostatistics [15].

## Results

### Patient demographics

The number of patients in each group and the proportion who underwent TAVR/SAVR are summarized in Supplemental Fig. 1. The characteristics of the TAVR and SAVR patients at baseline are summarized in Table 1. The patients’ mean age was significantly higher in the TAVR group (84.7 ± 4.3 years) compared to the SAVR group (74.3 ± 8.3 years, *p* < 0.001). There were more female patients in the TAVR group (71.8% vs. 41.2%, *p* < 0.001), while the body-mass index was lower in the TAVR group (22.1 ± 3.8 kg/m^2^ vs. 23.3 ± 3.9 kg/m^2^, *p* = 0.002). Furthermore, coronary artery diseases and peripheral vascular diseases were more prevalent in the SAVR group (*p* < 0.001 and *p* = 0.020, respectively). In contrast, the TAVR group tended to have cerebral vascular disease or carotid artery disease more frequently (*p* = 0.073). As TAVR is still prohibited in hemodialysis patients in Japan, the proportion of patients with renal dysfunction and on hemodialysis was higher in the SAVR group (*p* < 0.001). Bicuspid aortic valve was significantly more prevalent in the SAVR group (*p* < 0.001). The preoperative mean left ventricular ejection fraction was higher in the TAVR group (58.3 ± 11.2%) than in the SAVR group (55.0 ± 13.8%, *p* = 0.005). Emergency and urgent operations were more frequent in the SAVR group (*p* < 0.001), with concomitant or serial coronary revascularization being performed more frequently in the SAVR group (*p* < 0.001). The prostheses used in the TAVR and SAVR groups are summarized in Supplemental Table 1.

**Table 1:** Characteristics of the Patients at Baseline

Characteristics	TAVR	SAVR	*p* value
	(*N* = 238)	(*N* = 216)	
Age—year	84.7 ± 4.3	74.3 ± 8.3	< 0.001
Female sex—no. (%)	171 (71.8)	89 (41.2)	< 0.001
Body-mass index	22.1 ± 3.8	23.3 ± 3.9	0.002
STS-PROM	6.7 ± 4.6	5.7 ± 6.6	0.082
NYHA class III or IV—no. (%)	64 (26.9)	68 (31.5)	0.302
Coronary artery disease—no. (%)	35 (14.7)	74 (34.3)	< 0.001
Triple vessel disease and/or left main trunk disease—no (%)	3 (1.3)	28 (13.0)	< 0.001
Cerebral vascular disease/Carotid disease—no. (%)	63 (26.5)	41 (19.0)	0.073
Peripheral vascular disease—no. (%)	33 (13.9)	49 (22.7)	0.020
COPD—no. (%)	44 (18.5)	39 (18.1)	1.000
Creatinine > 2 mg/dl—no. (%)	3 (1.3)	43 (19.9)	< 0.001
Hemodialysis—no. (%)	0 (0)	34 (15.7)	< 0.001
Diabetes—no. (%)	78 (32.8)	77 (35.6)	0.553
Atrial fibrillation—no (%)	35 (14.7)	41 (19.0)	0.258
Previous cardiovascular surgery—no. (%)	12 (5.0)	12 (5.6)	0.836
Bicuspid aortic valve—no. (%)	6 (2.4)	42 (19.4)	< 0.001
Mitral insufficiency ≥ moderate—no. (%)	8 (3.4)	7 (3.3)	1.000
Left ventricular ejection fraction—%	58.3 ± 11.2	55.0 ± 13.8	0.005
Left ventricular ejection fraction < 30%—no. (%)	3 (1.3)	12 (5.6)	0.016
Emergent/Urgent operation—no. (%)	8 ( 3.4)	28 (13.0)	< 0.001
Concomitant CABG/TAVR + PCI—no. (%)	29 (12.2)	68 (31.5)	< 0.001
Institution: DMU—no. (%)	143 (60.1)	88 (40.7)	< 0.001

*TAVR* transcatheter aortic valve replacement, *SAVR* surgical aortic valve replacement, *STS-PROM* Society of Thoracic Surgery-Predicted Risk of Mortality, *NYHA* New York Heart Association, *COPD* chronic occlusive pulmonary disease, *CABG* coronary artery bypass grafting, *PCI* percutaneous coronary intervention, *DMU* Dokkyo Medical University

### Operative outcomes

The operative outcomes of the TAVR and SAVR groups are summarized in Table 2. Postoperatively, intra-aortic balloon pumping was required more frequently in the SAVR group (7.9%) than in the TAVR group (2.1%, *p* = 0.007). The SAVR group also experienced more intraoperative bleeding (*p* < 0.001), requiring more transfusions (*p* < 0.001). New-onset atrial fibrillation was observed in 29.6% of the SAVR patients and only 5.9% of the TAVR patients (*p* < 0.001). The majority of TAVR patients (77.7%) were extubated in the operating room, while none of the SAVR patients were extubated (*p* < 0.001). Intensive care unit stay was significantly longer in the SAVR group than in TAVR group (3.4 ± 3.9 days vs. 1.6 ± 3.9 days, *p* < 0.001). On the other hand, the TAVR group (9.2%) required more postoperative permanent pacemaker implantation than the SAVR patients (1.9%, *p* < 0.001). Peripheral vascular complications were observed only in TAVR patients (6.7% vs. 0%, *p* < 0.001).

**Table 2:** Clinical outcomes

Parameters	TAVR	SAVR	*p* value
	(*N* = 238)	(*N* = 216)	
Intraaortic balloon pump—no. (%)	5 (2.1)	17 (7.9)	0.007
Extracorporeal membrane oxygenation—no. (%)	4 (1.3)	3 (1.9)	0.713
Intraoperative bleeding—ml	173.9 ± 759.2	872.4 ± 617.3	< 0.001
Transfusion (red blood cell)—ml	371.5 ± 439.0	1111.7 ± 708.0	< 0.001
Reoperation for bleeding—no. (%)	8 ( 3.4)	16 ( 7.4)	0.061
New-onset atrial fibrillation—no. (%)	14 (5.9)	64 (29.6)	< 0.001
Permanent pacemaker implantation—no. (%)	22 (9.2)	4 (1.9)	< 0.001
Newly induced renal replacement therapy—no. (%)	4 (1.7)	6 (2.8)	0.529
Prosthetic valve endocarditis—no. (%)	1 (0.4)	4 (1.9)	0.195
Peripheral vascular complication—no. (%)	16 (6.7)	0 (0.0)	< 0.001
Extubation in operation room—no. (%)	185 (77.7)	0 (0.0)	< 0.001
Intubation time—hours	10.3 ± 52.3	18.5 ± 32.2	0.050
Intensive care unit stay—days	1.6 ± 3.9	3.4 ± 3.9	< 0.001
Echocardiographic findings			
Peak velocity through aortic valve—m/s	2.1 ± 0.5	2.4 ± 1.5	0.003
Mean pressure gradient—mmHg	9.9 ± 4.7	11.5 ± 4.8	< 0.001
Peak pressure gradient—mmHg	18.4 ± 8.4	21.2 ± 8.6	< 0.001
Effective orifice area index—cm^2^/m^2^	1.27 ± 0.35	1.06 ± 0.27	< 0.001
Effective orifice area index < 0.85 cm^2^/ m^2^—no. (%)	19 (8.0)	44 (20.4)	< 0.001
Effective orifice area index < 0.65 cm^2^/m^2^—no. (%)	2 (0.8)	7 (3.2)	0.092
≥ Trivial paravalvular leakage—no. (%)	205 (86.1)	16 (7.4)	< 0.001
≥ Mild paravalvular leakage—no. (%)	49 (20.6)	0 (0.0)	< 0.001

*TAVR* transcatheter aortic valve replacement, *SAVR* surgical aortic valve replacement

### Postoperative echocardiographic findings

The postoperative echocardiographic findings in the TAVR and SAVR groups are summarized in Table 2. Peak velocity through the prosthesis was significantly higher in patients with surgical valves (2.4 ± 1.5 m/s) than with transcatheter valves (2.1 ± 0.5 m/s, *p* = 0.003). Similarly, the mean and peak pressure gradients were also significantly higher in surgical valves (11.5 ± 4.8 and 21.2 ± 8.6 mmHg, respectively) than in transcatheter valves (9.9 ± 4.7 and 18.4 ± 8.4 mmHg, respectively, *p* < 0.001 for both comparisons). Meanwhile, the effective orifice area index was higher in transcatheter valves (1.27 ± 0.35 cm^2^/m^2^) than in surgical valves (1.06 ± 0.27 cm^2^/m^2^, *p* < 0.001). Patient-prosthesis mismatch (PPM) occurred more frequently in the SAVR group than in the TAVR group. Mild or more PPM (effective orifice area index < 0.85 cm^2^/m^2^) was observed in 20.4% of the SAVR group compared to 8.0% of the TAVR group (*p* < 0.001). Severe PPM (effective orifice area index < 0.65 cm^2^/m^2^) also tended to be seen more frequently in the SAVR group (3.2% vs. 0.8%, *p* = 0.092). In contrast, paravalvular leakage (PVL) was significantly more frequent in the TAVR group (86.1% vs. 7.4%, *p* < 0.001). Mild or greater PVL was also seen more frequently in the TAVR group (20.6%) than in the SAVR group (0%, *p* < 0.001).

### Mortality and morbidity

#### High-risk group

The baseline characteristics of the high-risk group patients are summarized in Supplemental Table 2-1A. The differences in the baseline characteristics between the TAVR and SAVR groups were similar to the overall patients. In brief, the patients in the TAVR group were older and more likely to be female patients. On the other hand, the SAVR group was associated with more coronary artery disease, peripheral vascular disease, renal dysfunction, emergent/urgent operations, and coronary procedures. Systolic heart function was significantly more impaired in the SAVR group (left ventricular ejection fraction 46.0 ± 15.0% vs. 52.0 ± 13.8%, *p* = 0.044), with the STS-PROM being significantly higher in SAVR (16.8 ± 8.2) than in TAVR (12.9 ± 5.2, *p* = 0.005). Furthermore, there were more patients classified as New York Heart Association functional class III or IV in the SAVR group (60.3%) than in the TAVR group (22.0%, *p* < 0.001).

**Fig. 1:**
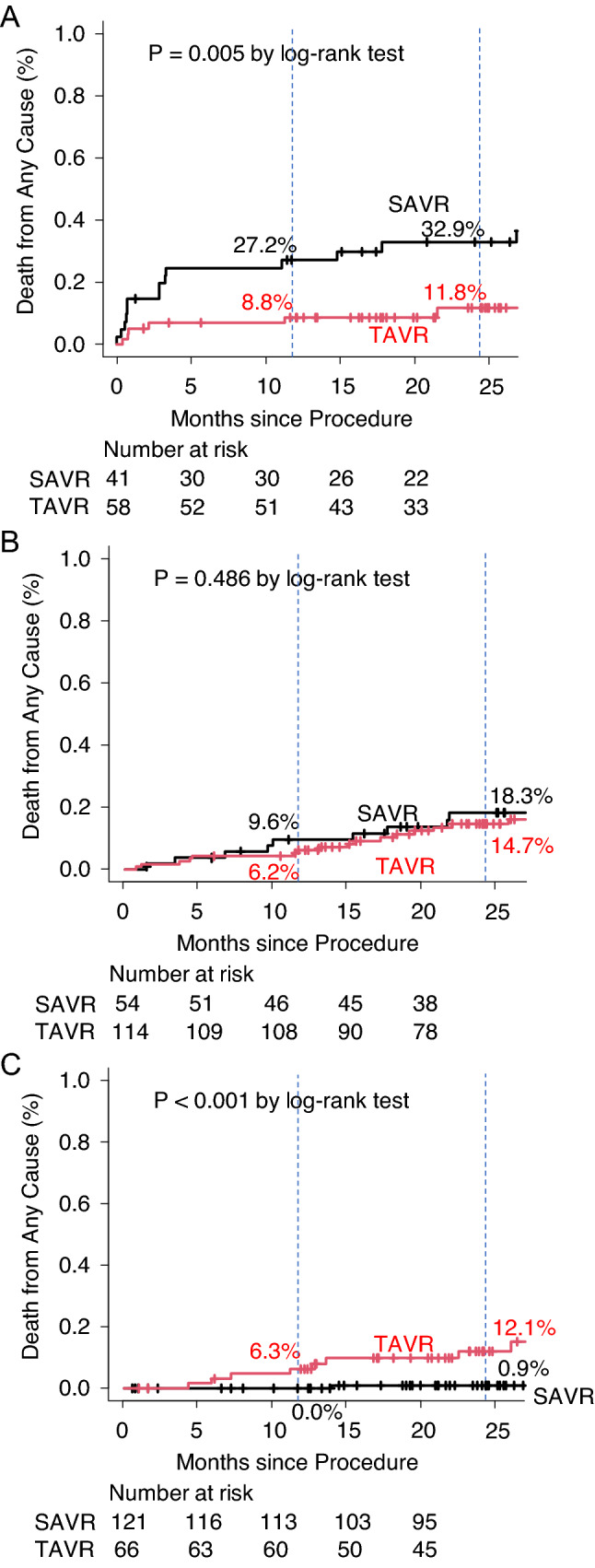
All-cause mortality in **A** high-risk group, **B** intermediate-risk group, and **C** low-risk group

The all-cause mortality at 1 and 2 years was 8.8% and 11.8% in the TAVR group and 27.2% and 32.9% in the SAVR group, respectively (*p* = 0.005) (Fig. 1A). The frequency of stroke and rehospitalization and the combined outcome of death, stroke, or rehospitalization were not significantly different between the groups (Supplemental Fig. 2A, 3A, 4A). Other clinical outcomes in the TAVR and SAVR groups are summarized in Supplemental Table 2-1B.

Among the preoperative factors, multivariable analysis using the Cox hazard model revealed that none of the factors, including operative procedures (TAVR or SAVR), were independent predictors of mortality (Supplemental Table 2-1C). Among the postoperative factors, the requirement of extracorporeal membrane oxygenation (ECMO) support (hazard ratio 51.97, *p* = 0.002), intraoperative bleeding (L, hazard ratio 1.54, *p* = 0.025), red blood cell transfusion (L, hazard ratio 2.17, *p* = 0.040), newly induced renal replacement therapy (hazard ratio 6.45, *p* = 0.004), and length of intensive care unit stay (days, hazard ratio 1.07, *p* = 0.008) were independent predictors of mortality (Supplemental Table 2-1D).

#### Intermediate-risk group

The baseline characteristics of the intermediate-risk group are summarized in Supplemental Table 2-2A. The differences in the TAVR and SAVR groups’ baseline characteristics were also similar to those of the overall patients.

There was no difference in all-cause mortality between the TAVR and SAVR groups (Fig. 1B). The incidence of stroke at 1 and 2 years was 2.6% and 2.6%, respectively, in the TAVR group and 9.6% and 11.8%, respectively, in the SAVR group (Supplemental Fig. 2B). The difference was statistically significant (*p* = 0.024). The frequency of rehospitalization did not differ between the groups (*p* = 0.172), and the combined outcome of death, stroke, or rehospitalization was also not significantly different between the groups (*p* = 0.192) (Supplemental Fig. 3B, 4B). The other clinical outcomes in the TAVR and SAVR groups are summarized in Supplemental Table 2-2B.

Among the preoperative factors, multivariable analysis using the Cox hazard model revealed that none of the factors, including operative procedures (TAVR or SAVR), were independent predictors of mortality (Supplemental Table 2-2C). Among the postoperative factors, the requirement of ECMO support (hazard ratio 11.73, *p* = 0.046) and prosthetic valve endocarditis (hazard ratio 13.88, *p* = 0.001) were independent predictors of mortality (Supplemental Table 2-2D).

#### Low-risk group

The baseline characteristics of the low-risk group are summarized in Supplemental Table 2-3A. The differences in the baseline characteristics between the TAVR and SAVR groups were somewhat different from those of the other two categories. Although the TAVR group had a higher mean age (81.8 ± 3.6 years vs. 71.6 ± 7.8 years, *p* < 0.001) and had more female patients (59.1% vs. 38.0%, *p* = 0.009), there was no significant difference in the prevalence of other comorbidities, except for cerebral vascular disease or carotid disease (TAVR vs. SAVR, 25.8% vs. 13.2%, *p* = 0.045). Therefore, due to the higher age in the TAVR group, STS-PROM was higher in this group (3.0 ± 0.6 vs. 2.2 ± 1.0, *p* < 0.001). Furthermore, the left ventricular ejection fraction was higher in the TAVR group (60.7 ± 9.5%) than in the SAVR group (56.9 ± 13.0%, *p* = 0.039) (Supplemental Table 2-3A).

No mortality occurred in either group for up to 4 months (Fig. 1C). The all-cause mortality at 1 and 2 years was 6.3% and 12.1%, respectively, in the TAVR group and 0% and 0.9%, respectively, in the SAVR group (Fig. 1C). The difference was statistically significant (*p* < 0.001). There was no significant difference in the frequency of stroke or rehospitalization, or the combined outcome of death, stroke, or rehospitalization between the groups (Supplemental Fig. 2C, 3C, 4C). The other clinical outcomes in the TAVR and SAVR groups are summarized in Supplemental Table 2-3B.

Among the preoperative factors, the multivariable analysis using the Cox hazard model revealed that only the operative procedure (TAVR) was the predictor of mortality (hazard ratio 20.89, *p* = 0.031) (Supplemental Table 2-3C). Among the postoperative factors, prosthetic valvular endocarditis (hazard ratio 48.27, *p* < 0.001) and mild or greater paravalvular leakage (hazard ratio 13.39, *p* = 0.007) were predictors of mortality (Supplemental Table 2-3D). The cause of death in in the low-risk group, along with the severity of postoperative paravalvular leakage, is listed in Supplemental Table 3.

The baseline characteristics of the low-risk group after the propensity score matching are summarized in Supplemental Table 4. There was no difference in the baseline characteristics between TAVR and SAVR groups. The all-cause mortality at 1 and 2 years was 4.6% and 21.4%, respectively, in the TAVR group and 0% and 0%, respectively, in the SAVR group (Supplemental Fig. 5). The difference was statistically significant (*p* = 0.042).

## Discussion

In 2020, the Japanese Circulation Society published a revision on the guidelines for the management of valvular heart disease [9]. In these guidelines, a clear age cutoff for the selection of TAVR or SAVR was not set. However, these offered an index of prioritization suggesting that TAVR should be utilized in patients aged ≥ 80 years, while SAVR should be used for patients aged < 75 years. The guidelines also emphasized the importance of the heart team in decision-making, including selecting a treatment approach. In our institutes, the selection of treatment (TAVR or SAVR) has always been performed through discussions within the heart team, and as a result, the mean patient age was significantly higher in the TAVR group than in the SAVR group, while the SAVR group had more comorbidities, such as coronary artery disease, peripheral vascular disease, renal dysfunction, and low left ventricular ejection fraction. Emergent/urgent operation and concomitant coronary intervention were also performed more frequently in the SAVR group than in the TAVR group. Given these differences, the most important findings in the present study were: (1) mortality in the high-risk group was as high as 27.2% at 1 year in the SAVR group, while it was significantly lower in the TAVR group (8.8% at 1 year); (2) there was no significant difference in mortality between the TAVR and SAVR groups in the intermediate-risk group; (3) mortality in the low-risk group was significantly higher in the TAVR group than in the SAVR group, and the procedure (TAVR) was a significant predictor of mortality; (4) TAVR was less invasive compared to SAVR, as seen in the decrease in bleeding, blood transfusion requirement, new-onset atrial fibrillation, and intensive care unit stay; (5) and the performance of transcatheter valves was better compared to surgical valves, with lower peak velocity and pressure gradient, higher effective orifice area index, and less frequent PPM.

The results of mortality and morbidity analyses in the high-risk group in the present study were compatible with those of industry-driven RCTs since TAVR was comparable with, or even superior to, SAVR [1–3]. In the PARTNER trial, which compared TAVR and SAVR in high-risk patients [2], the 1-year mortality of the SAVR arm was 26.8%, similar to that of the SAVR group in our study (27.2%). Furthermore, in the said trial, the patients’ age and STS-PROM scores in the TAVR group were 83.6 ± 6.8 years and 11.8 ± 3.3, respectively, while the 1-year mortality was 24.2% [2]. In contrast, the 1-year mortality of the high-risk TAVR group in the present study was much lower (8.8%), although the age (86.5 ± 4.9 years) and STS-PROM (12.9 ± 5.2) were higher.

In 2016 and 2017, the results of two industry-driven RCTs comparing TAVR and SAVR in intermediate-risk AS patients were published [4, 5]. Both studies revealed the noninferiority of TAVR to SAVR. The intermediate-risk group’s mortality and morbidity analysis results in the present study were also compatible with these two RCTs. Therefore, it is indisputable that TAVR should be the first choice of treatment for severe AS in high- and intermediate-risk patients.

On the other hand, the mortality and morbidity analysis results in the low-risk group in the present study slightly varied from those of industry-driven RCTs [6, 7]. In the present study, postoperative mortality was significantly lower in the SAVR group than in the TAVR group. Even though the patients were older and the frequency of cerebral vascular disease/carotid disease and preoperative STS-PROM were higher in the TAVR group, these preoperative factors were not detected as significant risk factors of mortality in the multivariable Cox hazard model analysis. Only the preoperative factor of “procedure = TAVR” was detected as a significant risk factor for mortality. The result was the same even after the propensity score matching. As for the analysis of postoperative factors, mild or greater paravalvular leakage was detected as a significant risk factor for mortality. Over 80% of the TAVR patients had trivial or greater paravalvular leakage, with 18.2% having mild or greater paravalvular leakage, possibly explaining why TAVR was a risk factor for mortality. It is widely known that moderate and severe aortic regurgitation is associated with increased mortality [4, 16].

In the PARTNER 3 trial, the 1-year mortality of the SAVR group, which had a mean age of 73.6 ± 6.1 years and a mean STS-PROM of 1.9 ± 0.6, was 2.5% [6]. In contrast, in the low-risk SAVR group in the present study, which had a mean age of 71.6 ± 7.8 years and a mean STS-PROM of 2.2 ± 1.0, the 1- and 2-year mortalities were 0% and 0.9%, respectively. In our “real-world” experiences, patients under 75 years without any comorbidities that increase the STS-PROM score seldom die early after aortic valve replacement surgery. With a 1-year mortality of 2.5%, the PARTNER 3 trial could not dispel the impression that the outcomes in the SAVR arm were “sub-optimal” [13]. Furthermore, in the said trial, 5 patients died within 30 days, with 3 dying due to “pulseless electrical activity arrest,” 1 due to respiratory failure, and 1 due to sepsis. One patient also died due to “failure to wean from extracorporeal membrane oxygenation” on day 31 [6]. One can only wonder what happened to these six patients intraoperatively.

### Study limitations

The present study had several limitations, including its retrospective nature. As stated above, the indications for TAVR or SAVR were determined through discussion within the heart team and the preoperative patient characteristics were significantly different between the TAVR and SAVR groups. In this study, risk stratification was done only by the STS-PROM, although patients underwent each procedure due to multiple reasons. For example, some patients underwent SAVR despite high STS-PROM because of hemodialysis, complex coronary artery disease, etc., while other patients underwent TAVR despite low STS-PROM because of frailty and so on. Therefore, it was impossible to compare the results of the present study and previously published industry-driven RCTs. Similar to the industry-driven RCTs, the most important data that the present study failed to show was the patients’ long-term outcomes, including data on prosthetic valve deterioration. The data would be essential in deciding whether the indication for TAVR should be expanded to young and low-risk patients. Hopefully, future studies will help clarify this.

## Conclusions

We have demonstrated that the clinical outcomes of TAVR were non-inferior or even superior to those of SAVR in high- and intermediate-risk patients with severe AS, and the priority of TAVR in these patient populations is indisputable. On the other hand, indications for TAVR in low-risk AS patients should be carefully discussed, because mild or more PVL was a risk factor for short-term mortality.

## Supplementary Information

Below is the link to the electronic supplementary material.

Supplementary file1 (DOCX 18 KB)

Supplementary file2 (DOCX 73 KB)

Supplementary file3 (DOCX 18 KB)

Supplementary file4 (DOCX 25 KB)

Supplementary file5 (PPTX 49 KB)

Supplementary file6 (PPTX 70 KB)

Supplementary file7 (PPTX 72 KB)

Supplementary file8 (PPTX 70 KB)

Supplementary file9 (PPTX 41 KB)

Supplementary file10 (DOCX 14 KB)
