# Supplementary material for: Contemporary short-term outcomes of surgery for aortic stenosis: transcatheter vs. surgical aortic valve replacement
Source: Gen Thorac Cardiovasc Surg. 2021 Jun 22;70(2):124–31. doi: 10.1007/s11748-021-01672-8 (PMC8817997; doi:10.1007/s11748-021-01672-8)
Supplement: Supplementary file 1 — Supplementary file1 (DOCX 18 KB) [file 11748_2021_1672_MOESM1_ESM.docx]

**Supplemental Table 1. Prosthesis used in this study**

**Size (mm)**

**Prosthesis 19 20 21 22 23 25 26 27 29**

**TAVR**

Sapien XT - 1 - - 23 - 12 - 1

Sapien 3 - 6 - - 95 - 40 - 11

CoreValve - 0 - - 0 - 1 - 1

CoreValve EVOLUTE R - 0 - - 4 - 19 - 13

CoreValve EVOLUTE PRO - 0 - - 1 - 6 - 5

**SAVR**

**Biological**

Magna Ease 16 - 29 - 14 8 - 0 0

INSPIRIS RESILIA 3 - 2 - 7 0 - 0 0

Carpentier-Edwards PERIMOUNT 0 - 2 - 3 1 - 1 0

Crown PRT 4 - 34 - 43 8 - 0 0

SOLO SMART 1 - 6 - 3 0 - 0 0

Mitroflow 2 - 0 - 0 0 - 0 0

Trifecta 1 - 4 - 1 0 - 0 0

Mosaic Ultra 1 - 0 - 2 0 - 0 0

AVALUS 1 - 0 - 3 1 - 1 0

**Mechanical**

SJM Regent 3 - 3 - 2 0 - 0 0

ATS - 2 - 2 - - 0 - -

On-X 0 - 0 - 2 0 - 0 0
