# Supplementary material for: Contemporary short-term outcomes of surgery for aortic stenosis: transcatheter vs. surgical aortic valve replacement
Source: Gen Thorac Cardiovasc Surg. 2021 Jun 22;70(2):124–31. doi: 10.1007/s11748-021-01672-8 (PMC8817997; doi:10.1007/s11748-021-01672-8)
Supplement: Supplementary file 2 — Supplementary file2 (DOCX 73 KB) [file 11748_2021_1672_MOESM2_ESM.docx]

**Supplemental Table 2-1A. Characteristics of the High-risk Group Patients at Baseline.**

**TAVR SAVR**

**Characteristics (N = 58) (N = 41) p value**

Age --- year 86.5 ± 4.9 78.6 ± 8.4 <0.001

Female sex --- no. (%) 45 (77.6) 12 (29.3) <0.001

Body-mass index 21.6 ± 3.5 21.5 ± 3.3 0.903

STS-PROM 12.9 ± 5.2 16.8 ± 8.2 0.005

NYHA class III or IV --- no. (%) 23 (22.0) 32 (60.3) <0.001

Coronary artery disease --- no. (%) 12 (20.7) 24 (58.5) <0.001

Triple vessel disease and/or left main trunk disease --- no (%) 2 ( 3.4) 12 (29.3) <0.001

Cerebral vascular disease / Carotid disease --- no. (%) 23 (39.7) 10 (24.4) 0.133

Peripheral vascular disease --- no. (%) 18 (31.0) 23 (56.1) 0.014

COPD --- no. (%) 15 (25.9) 9 (22.0) 0.812

Creatinine > 2 mg/dl --- no. (%) 3 ( 5.2) 22 (53.7) <0.001

Hemodialysis --- no. (%) 0 ( 0) 17 (41.5) <0.001

Diabetes --- no. (%) 22 (37.9) 18 (43.9) 0.678

Atrial fibrillation --- no (%) 15 (25.9) 14 (34.1) 0.381

Previous cardiovascular surgery --- no. (%) 8 (13.8) 3 ( 7.3) 0.354

Bicuspid aortic valve --- no. (%) 2 ( 3.4) 3 ( 7.3) 0.646

Mitral insufficiency ≥ moderate --- no. (%) 3 ( 5.2) 3 ( 7.3) 0.690

Left ventricular ejection fraction --- % 52.0 ± 13.8 46.0 ± 15.0 0.044

Left ventricular ejection fraction < 30 ---no. (%) 2 ( 3.4) 6 (14.6) 0.063

Emergent / Urgent operation --- no. (%) 8 (13.8) 20 (48.8) <0.001

Concomitant CABG / TAVR + PCI --- no. (%) 9 (15.5) 22 (53.7) <0.001

Institution: DMU --- no. (%) 36 (62.1) 17 (41.5) 0.065

TAVR: transcatheter aortic valve replacement, SAVR: surgical aortic valve replacement, STS-PROM: Society of Thoracic Surgery-Predicted Risk of Mortality, NYHA: New York Heart Association, COPD: chronic occlusive pulmonary disease, CABG: coronary artery bypass grafting, PCI: percutaneous coronary intervention, DMU: Dokkyo Medical University

**Supplemental Table 2-1B. Clinical Outcomes of the High-risk Group**

**TAVR SAVR**

**Parameters (N = 58) (N = 41) p value**

Intraaortic balloon pump --- no. (%) 3 ( 5.2) 10 (24.4) 0.007

Extracorporeal membrane oxygenation --- no. (%) 0 ( 0.0) 4 ( 9.8) 0.027

Intraoperative bleeding --- ml 238.3 ± 1300.3 1067.5 ± 620.3 <0.001

Transfusion (red blood cell) --- ml 416.4 ± 418.0 1495.6 ± 712.1 <0.001

Reoperation for bleeding --- no. (%) 1 ( 1.7) 5 (12.2) 0.079

Newly onset atrial fibrillation --- no. (%) 4 ( 6.9) 13 (31.7) 0.002

Permanent pacemaker implantation --- no. (%) 7 (12.1) 1 ( 2.4) 0.135

Newly induced renal replacement therapy --- no. (%) 3 ( 5.2) 3 ( 7.3) 0.690

Prosthetic valve endocarditis --- no. (%) 0 ( 0.0) 0 ( 0.0) -

Peripheral vascular complication --- no. (%) 4 ( 6.0) 0 ( 0.0) 0.140

Extubation in operation room --- no. (%) 37 (63.8) 0 ( 0.0) <0.001

Intubation time --- hours 32.6 ± 100.4 39.9 ± 45.3 0.681

Intensive care unit stay --- days 3.2 ± 6.3 7.2 ± 7.5 0.006

Echocardiographic findings

Peak velocity through aortic valve --- m/s 2.0 ± 0.5 2.2 ± 0.5 0.009

Mean pressure gradient --- mmHg 8.8 ± 4.6 11.1 ± 4.6 0.021

Peak pressure gradient --- mmHg 15.8 ± 7.9 20.8 ± 8.3 0.004

Effective orifice area index --- cm^2^/m^2^ 1.18 ± 0.32 1.03 ± 0.37 0.064

Effective orifice area index < 0.85 cm^2^/ m^2^ --- no. (%) 8 (13.8) 8 (19.5) 0.404

Effective orifice area index < 0.65 cm^2^/ m^2^ --- no. (%) 1 ( 1.5) 3 ( 7.3) 0.304

≥Trivial paravalvular leakage --- no. (%) 50 (86.2) 4 ( 9.8) <0.001

≥Mild paravalvular leakage --- no. (%) 6 (10.3) 0 ( 0.0) 0.040

TAVR: transcatheter aortic valve replacement, SAVR: surgical aortic valve replacement

**Supplemental Table 2-1C. Preoperative Risk Factors for Death in the High-risk Group (Cox Hazard Model)**

**Univariate Multivariable**

**Parameters p value p value Hazard ratio (95%CI)**

Age 0.035 0.493 1.02 (0.96 – 1.09)

Female sex 0.002 0.050 0.34 (0.12 – 1.00)

Body-mass index 0.173

STS-PROM 0.003 0.188 1.04 (0.98 – 1.09)

NYHA class III or IV 0.010 0.137 2.65 (0.73 – 9.54)

Coronary artery disease 0.027 0.911 1.10 (0.23– 5.17)

Triple vessel disease and/or left main trunk disease 0.436

Cerebral vascular disease / Carotid disease 0.149

Peripheral vascular disease 0.050 0.903 1.07 (0.38 – 3.03)

COPD 0.986

Creatinine > 2 mg/dl 0.125

Hemodialysis 0.069 0.258 2.06 (0.59 – 7.24)

Diabetes 0.508

Atrial fibrillation 0.218

Previous cardiovascular surgery 0.215

Bicuspid aortic valve 0.927

Mitral insufficiency ≥ moderate 0.751

Left ventricular ejection fraction 0.084 0.886 1.00 (0.97 – 1.04)

Left ventricular ejection fraction < 30% 0.102

Emergent / Urgent operation 0.253

Concomitant CABG / TAVR + PCI 0.007 0.772 1.26 (0.27 – 5.98)

TAVR 0.008 0.982 0.99 (0.29 – 3.38)

Institution: DMU 0.327

STS-PROM: Society of Thoracic Surgery-Predicted Risk of Mortality, NYHA: New York Heart Association, COPD: chronic occlusive pulmonary disease, CABG: coronary artery bypass grafting, PCI: percutaneous coronary intervention, TAVR: transcatheter aortic valve replacement, DMU: Dokkyo Medical University

**Supplemental Table 2-1D. Postoperative Risk Factors for Death in the High-risk Group (Cox Hazard Model)**

**Univariate Multivariable**

**Parameters p value p value Hazard ratio (95%CI)**

Intraaortic balloon pump 0.001 0.847 0.89 (0.27 – 2.96)

Extracorporeal membrane oxygenation <0.001 0.002 51.97 (4.04 – 669.30)

Intraoperative bleeding (L) <0.001 0.025 1.54 (1.06 – 2.24)

Transfusion (red blood cell) (L) <0.001 0.040 2.17 (1.04 – 4.57)

Reoperation for bleeding 0.033 0.745 0.73 (0.11 – 4.93)

Newly onset atrial fibrillation 0.670

Permanent pacemaker implantation 0.390

Newly induced renal replacement therapy 0.002 0.004 6.45 (1.83 – 22.72)

Prosthetic valve endocarditis (no event)

Peripheral vascular complication 0.775

Intubation time (hr) 0.121

Intensive care unit stay (days) <0.001 0.008 1.07 (1.02 – 1.12)

Echocardiographic findings

Peak velocity through aortic valve (m/sec) 0.899

Mean pressure gradient (mmHg) 0.612

Peak pressure gradient (mmHg) 0.828

Effective orifice area index (cm^2^/m^2^) 0.363

≥Trivial paravalvular leakage 0.017 0.659 1.36 (0.35 – 5.35)

≥Mild paravalvular leakage 0.998

**Supplemental Table 2-2A. Characteristics of the Intermediate-risk Group Patients at Baseline.**

**TAVR SAVR**

**Characteristics (N = 114) (N = 54) p value**

Age --- year 85.4 ± 3.6 76.9 ± 7.3 <0.001

Female sex --- no. (%) 87 (76.3) 31 (57.4) <0.001

Body-mass index 22.0 ± 3.1 22.6 ± 4.0 0.303

STS-PROM 5.6 ± 1.1 5.4 ± 1.2 0.230

NYHA class III or IV --- no. (%) 28 (24.6) 18 (33.3) 0.268

Coronary artery disease --- no. (%) 15 (13.2) 22 (40.7) <0.001

Triple vessel disease and/or left main trunk disease --- no (%) 1 ( 0.9) 11 (20.4) <0.001

Cerebral vascular disease / Carotid disease --- no. (%) 23 (20.2) 15 (27.8) 0.324

Peripheral vascular disease --- no. (%) 12 (10.5) 15 (27.8) 0.007

COPD --- no. (%) 22 (19.3) 10 (18.5) 1

Creatinine > 2 mg/dl --- no. (%) 0 ( 0) 18 (33.3) <0.001

Hemodialysis --- no. (%) 0 ( 0) 16 (29.6) <0.001

Diabetes --- no. (%) 36 (31.6) 18 (33.3) 0.860

Atrial fibrillation --- no (%) 14 (12.3) 11 (20.4) 0.173

Previous cardiovascular surgery --- no. (%) 3 ( 2.6) 2 ( 3.7) 0.657

Bicuspid aortic valve --- no. (%) 3 ( 2.6) 2 ( 3.7) 0.657

Mitral insufficiency ≥ moderate --- no. (%) 3 ( 2.6) 2 ( 3.7) 0.657

Left ventricular ejection fraction --- % 60.1 ± 9.4 57.5 ± 11.7 0.122

Left ventricular ejection fraction < 30 ---no. (%) 0 ( 0) 2 (3.7) 0.102

Emergent / Urgent operation --- no. (%) 0 ( 0) 5 ( 9.3) 0.003

Concomitant CABG / TAVR + PCI --- no. (%) 12 (10.5) 21 (38.9) <0.001

Institution: DMU --- no. (%) 70 (61.4) 19 (35.2) 0.002

TAVR: transcatheter aortic valve replacement, SAVR: surgical aortic valve replacement, STS-PROM: Society of Thoracic Surgery-Predicted Risk of Mortality, NYHA: New York Heart Association, COPD: chronic occlusive pulmonary disease, CABG: coronary artery bypass grafting, PCI: percutaneous coronary intervention, DMU: Dokkyo Medical University

**Supplemental Table 2-2B. Clinical Outcomes of the Intermediate-risk Group**

**TAVR SAVR**

**Parameters (N = 114) (N = 54) p value**

Intraaortic balloon pump --- no. (%) 0 ( 0.0) 5 ( 9.3) 0.003

Extracorporeal membrane oxygenation --- no. (%) 1 ( 0.9) 0 ( 0.0) 1

Intraoperative bleeding --- ml 189.1 ± 565.8 987.4 ± 743.1 <0.001

Transfusion (red blood cell) --- ml 375.8 ± 491.1 1446.7 ± 641.3 <0.001

Reoperation for bleeding --- no. (%) 5 ( 4.4) 4 ( 7.4) 0.471

Newly onset atrial fibrillation --- no. (%) 6 ( 5.3) 12 (22.2) 0.002

Permanent pacemaker implantation --- no. (%) 11 ( 9.6) 1 ( 1.9) 0.106

Newly induced renal replacement therapy --- no. (%) 1 ( 0.9) 2 ( 3.7) 0.242

Prosthetic valve endocarditis --- no. (%) 0 ( 0.0) 2 ( 3.7) 0.102

Peripheral vascular complication --- no. (%) 8 ( 7.0) 0 ( 0.0) 0.056

Extubation in operation room --- no. (%) 94 (82.5) 0 ( 0.0) <0.001

Intubation time --- hours 1.4 ± 4.8 20.4 ± 33.4 <0.001

Intensive care unit stay --- days 1.1 ± 3.0 3.3 ± 2.0 <0.001

Echocardiographic findings

Peak velocity through aortic valve --- m/s 2.1 ± 0.5 2.3 ± 0.6 0.083

Mean pressure gradient --- mmHg 10.1 ± 4.8 11.9 ± 5.6 0.033

Peak pressure gradient --- mmHg 19.0 ± 8.7 21.8 ± 10.0 0.068

Effective orifice area index --- cm^2^/m^2^ 1.32 ± 0.35 1.09 ± 0.28 <0.001

Effective orifice area index < 0.85 cm^2^/ m^2^ --- no. (%) 6 ( 5.4) 12 (23.1) 0.002

Effective orifice area index < 0.65 cm^2^/ m^2^ --- no. (%) 0 ( 0.0) 3 ( 5.6) 0.032

≥Trivial paravalvular leakage --- no. (%) 98 (86.0) 2 ( 3.7) <0.001

≥Mild paravalvular leakage --- no. (%) 31 (27.2) 0 ( 0.0) <0.001

TAVR: transcatheter aortic valve replacement, SAVR: surgical aortic valve replacement

**Supplemental Table 2-2C. Preoperative Risk Factors for Death in the Intermediate-risk Group (Cox Hazard Model)**

**Univariate Multivariable**

**Parameters p value p value Hazard ratio (95%CI)**

Age 0.070 0.480 0.98 (0.93 – 1.04)

Female sex 0.037 0.236 0.60 (0.26 – 1.49)

Body-mass index 0.201

STS-PROM 0.787

NYHA class III or IV 0.617

Coronary artery disease 0.152

Triple vessel disease and/or left main trunk disease 0.414

Cerebral vascular disease / Carotid disease 0.576

Peripheral vascular disease 0.278

COPD 0.423

Creatinine > 2 mg/dl 0.102

Hemodialysis 0.062 0.564 1.45 (0.41 – 5.09)

Diabetes 0.315

Atrial fibrillation 0.022 0.102 2.08 (0.87 – 5.01)

Previous cardiovascular surgery 0.890

Bicuspid aortic valve 0.241

Mitral insufficiency ≥ moderate 0.092 0.363 2.10 (0.43 – 10.30)

Left ventricular ejection fraction 0.615

Left ventricular ejection fraction < 30% 0.997

Emergent / Urgent operation 0.821

Concomitant CABG / TAVR + PCI 0.867

TAVR 0.488

Institution: DMU 0.545

STS-PROM: Society of Thoracic Surgery-Predicted Risk of Mortality, NYHA: New York Heart Association, COPD: chronic occlusive pulmonary disease, CABG: coronary artery bypass grafting, PCI: percutaneous coronary intervention, TAVR: transcatheter aortic valve replacement, DMU: Dokkyo Medical University

**Supplemental Table 2-2D. Postoperative Risk Factors for Death in the Intermediate-risk Group (Cox Hazard Model)**

**Univariate Multivariable**

**Parameters p value p value Hazard ratio (95%CI)**

Intraaortic balloon pump 0.092 0.360 2.21 (0.41 – 12.02)

Extracorporeal membrane oxygenation 0.010 0.046 11.73 (1.05 – 131.60)

Intraoperative bleeding (L) 0.862

Transfusion (red blood cell) (L) 0.503

Reoperation for bleeding 0.721

Newly onset atrial fibrillation 0.473

Permanent pacemaker implantation 0.522

Newly induced renal replacement therapy 0.431

Prosthetic valve endocarditis <0.001 0.001 13.88 (2.79 – 68.98)

Peripheral vascular complication 0.822

Intubation time (hr) 0.321

Intensive care unit stay (days) 0.002 0.135 1.10 (0.97 – 1.24)

Echocardiographic findings

Peak velocity through aortic valve (m/sec) 0.200

Mean pressure gradient (mmHg) 0.273

Peak pressure gradient (mmHg) 0.436

Effective orifice area index (cm^2^/m^2^) 0.576

≥Trivial paravalvular leakage 0.894

≥Mild paravalvular leakage 0.907

**Supplemental Table 2-3A. Characteristics of the Low-risk Group Patients at Baseline.**

**TAVR SAVR**

**Characteristics (N = 66) (N = 121) p value**

Age --- year 81.8 ± 3.6 71.6 ± 7.8 <0.001

Female sex --- no. (%) 39 (59.1) 46 (38.0) 0.009

Body-mass index 22.8 ± 4.9 24.1 ± 3.8 0.035

STS-PROM 3.0 ± 0.6 2.2 ± 1.0 <0.001

NYHA class III or IV --- no. (%) 13 (19.7) 18 (14.9) 0.416

Coronary artery disease --- no. (%) 8 (12.1) 28 (23.1) 0.082

Triple vessel disease and/or left main trunk disease --- no (%) 0 ( 0) 5 (4.1) 0.163

Cerebral vascular disease / Carotid disease --- no. (%) 17 (25.8) 16 (13.2) 0.045

Peripheral vascular disease --- no. (%) 3 ( 4.5) 11 ( 9.1) 0.385

COPD --- no. (%) 7 (10.6) 20 (16.5) 0.384

Creatinine > 2 mg/dl --- no. (%) 0 ( 0) 3 ( 2.5) 0.553

Hemodialysis --- no. (%) 0 ( 0) 1 ( 0.8) 1.000

Diabetes --- no. (%) 20 (30.3) 41 (33.9) 0.744

Atrial fibrillation --- no (%) 6 ( 9.1) 16 (13.2) 0.482

Previous cardiovascular surgery --- no. (%) 1 ( 1.5) 7 ( 5.8) 0.264

Bicuspid aortic valve --- no. (%) 1 (1.5) 37 (30.6) <0.001

Mitral insufficiency ≥ moderate --- no. (%) 2 (3.0) 2 ( 1.7) 0.615

Left ventricular ejection fraction --- % 60.7 ± 9.5 56.9 ± 13.0 0.039

Left ventricular ejection fraction < 30 ---no. (%) 1 (1.5) 4 ( 3.3) 0.658

Emergent / Urgent operation --- no. (%) 0 ( 0) 3 ( 2.5) 0.553

Concomitant CABG / TAVR + PCI --- no. (%) 8 (12.1) 25 (20.7) 0.164

Institution: DMU --- no. (%) 37 (56.1) 52 (43.0) 0.092

TAVR: transcatheter aortic valve replacement, SAVR: surgical aortic valve replacement, STS-PROM: Society of Thoracic Surgery-Predicted Risk of Mortality, NYHA: New York Heart Association, COPD: chronic occlusive pulmonary disease, CABG: coronary artery bypass grafting, PCI: percutaneous coronary intervention, DMU: Dokkyo Medical University

**Supplemental Table 2-3B. Clinical Outcomes of the Low-risk Group**

**TAVR SAVR**

**Parameters (N = 66) (N = 121) p value**

Intraaortic balloon pump --- no. (%) 1 ( 1.5) 3 ( 2.5) 1.000

Extracorporeal membrane oxygenation --- no. (%) 1 ( 1.5) 0 ( 0.0) 0.354

Intraoperative bleeding --- ml 87.3 ± 146.7 754.0 ± 526.2 <0.001

Transfusion (red blood cell) --- ml 324.5 ± 355.2 832.1 ± 606.5 <0.001

Reoperation for bleeding --- no. (%) 2 ( 3.0) 7 ( 5.8) 0.496

Newly onset atrial fibrillation --- no. (%) 4 ( 6.1) 39 (32.2) <0.001

Permanent pacemaker implantation --- no. (%) 4 ( 6.1) 2 ( 1.7) 0.187

Newly induced renal replacement therapy --- no. (%) 0 ( 0.0) 1 ( 0.8) 1.000

Prosthetic valve endocarditis --- no. (%) 1 ( 1.5) 2 ( 1.6) 1.000

Peripheral vascular complication --- no. (%) 4 ( 6.1) 0 ( 0.0) 0.015

Extubation in operation room --- no. (%) 54 (81.8) 0 ( 0.0) <0.001

Intubation time --- hours 6.4 ± 24.0 11.1 ± 23.3 0.194

Intensive care unit stay --- days 1.1 ± 1.6 2.4 ± 1.8 <0.001

Echocardiographic findings

Peak velocity through aortic valve --- m/s 2.2 ± 0.4 2.3 ± 0.4 0.202

Mean pressure gradient --- mmHg 10.6 ± 4.6 11.4 ± 4.5 0.257

Peak pressure gradient --- mmHg 19.7 ± 7.9 21.0 ± 8.1 0.281

Effective orifice area index --- cm^2^/m^2^ 1.27 ± 0.37 1.06 ± 0.23 <0.001

Effective orifice area index < 0.85 cm^2^/ m^2^ --- no. (%) 5 ( 7.6) 24 (19.8) 0.034

Effective orifice area index < 0.65 cm^2^/ m^2^ --- no. (%) 1 ( 1.5) 1 ( 0.8) 1.000

≥Trivial paravalvular leakage --- no. (%) 57 (86.4) 10 ( 8.3) <0.001

≥Mild paravalvular leakage --- no. (%) 12 (18.2) 0 ( 0.0) <0.001

TAVR: transcatheter aortic valve replacement, SAVR: surgical aortic valve replacement

**Supplemental Table 2-3C. Preoperative Risk Factors for Death in the Low-risk Group (Cox Hazard Model)**

**Univariate Multivariable**

**Parameters p value p value Hazard ratio (95%CI)**

Age 0.084 0.369 0.93 (0.79 – 1.09)

Female sex 0.206

Body-mass index 0.958

STS-PROM 0.084 0.380 1.63 (0.55 – 4.81)

NYHA class III or IV 0.607

Coronary artery disease 0.525

Triple vessel disease and/or left main trunk disease 0.999

Cerebral vascular disease / Carotid disease 0.210

Peripheral vascular disease 0.672

COPD 0.998

Creatinine > 2 mg/dl 0.998

Hemodialysis 0.998

Diabetes 0.555

Atrial fibrillation 0.348

Previous cardiovascular surgery 0.998

Bicuspid aortic valve 0.506

Mitral insufficiency ≥ moderate 0.110

Left ventricular ejection fraction 0.077 0.149 1.06 (0.98 – 1.16)

Left ventricular ejection fraction < 30% 0.998

Emergent / Urgent operation 0.998

Concomitant CABG / TAVR + PCI 0.588

TAVR 0.001 0.031 20.89 (1.32 – 332.70)

Institution: DMU 0.407

STS-PROM: Society of Thoracic Surgery-Predicted Risk of Mortality, NYHA: New York Heart Association, COPD: chronic occlusive pulmonary disease, CABG: coronary artery bypass grafting, PCI: percutaneous coronary intervention, TAVR: transcatheter aortic valve replacement, DMU: Dokkyo Medical University

**Supplemental Table 2-3D. Postoperative Risk Factors for Death in the Low-risk Group (Cox Hazard Model)**

**Univariate Multivariable**

**Parameters p value p value Hazard ratio (95%CI)**

Intraaortic balloon pump 0.999

Extracorporeal membrane oxygenation 1.000

Intraoperative bleeding (L) 0.162

Transfusion (red blood cell) (L) 0.332

Reoperation for bleeding 0.998

Newly onset atrial fibrillation 0.998

Permanent pacemaker implantation 0.999

Newly induced renal replacement therapy 0.999

Prosthetic valve endocarditis <0.001 <0.001 48.27 (6.54 – 356.40)

Peripheral vascular complication 0.072 0.999 0.00 (0.00 – 0.00)

Intubation time (hr) 0.695

Intensive care unit stay (days) 0.581

Echocardiographic findings

Peak velocity through aortic valve (m/sec) 0.910

Mean pressure gradient (mmHg) 0.687

Peak pressure gradient (mmHg) 0.858

Effective orifice area index (cm^2^/m^2^) 0.035 0.563 1.79 (0.25 – 12.82)

≥Trivial paravalvular leakage 0.009 0.174 5.00 (0.49 – 50.78)

≥Mild paravalvular leakage <0.001 0.007 13.39 (2.05 – 87.64)

**Supplemental Table 3. Baseline Patient Characteristics (Low-risk Group) after Propensity Score Matching.**

**TAVR SAVR**

**Characteristics (N = 23) (N = 23) p value**

Age --- year 79.3 ± 3.6 79.0 ± 3.5 0.742

Female sex --- no. (%) 12 (52.2) 11 (47.8) 1.000

Body-mass index 22.2 ± 4.2 23.1 ± 3.3 0.458

STS-PROM 2.5 ± 0.5 2.7 ± 0.9 0.235

NYHA class III or IV --- no. (%) 4 (17.4) 2 ( 8.7) 0.665

Coronary artery disease --- no. (%) 2 ( 8.7) 4 (17.4) 0.665

Triple vessel disease and/or left main trunk disease --- no (%) 0 ( 0) 0 ( 0) -

Cerebral vascular disease / Carotid disease --- no. (%) 5 (21.7) 6 (26.1) 1.000

Peripheral vascular disease --- no. (%) 1 ( 4.3) 1 ( 4.3) 1.000

COPD --- no. (%) 3 (13.0) 4 (17.4) 1.000

creatinine > 2 mg/dl --- no. (%) 0 ( 0) 1 ( 4.3) 1.000

Hemodialysis --- no. (%) 0 ( 0) 0 ( 0) -

Diabetes --- no. (%) 8 (34.8) 6 (26.1) 0.749

Atrial fibrillation --- no (%) 1 ( 4.3) 4 (17.4) 0.346

Previous cardiovascular surgery --- no. (%) 0 ( 0) 0 ( 0) -

Bicuspid aortic valve --- no. (%) 1 ( 4.3) 0 ( 0) 1.000

Mitral insufficiency ≥ moderate --- no. (%) 1 ( 4.3) 0 ( 0) 1.000

Left ventricular ejection fraction --- % 60.1 ± 11.2 60.2 ± 8.1 0.992

Left ventricular ejection fraction < 30 ---no. (%) 1 ( 4.3) 0 ( 0) 1

Emergent / Urgent operation --- no. (%) 0 ( 0) 0 ( 0) -

Concomitant CABG / TAVR + PCI --- no. (%) 2 (8.7) 4 (17.4) 0.665

Institution: DMU --- no. (%) 15 (65.2) 9 (39.1) 0.139

TAVR: transcatheter aortic valve replacement, SAVR: surgical aortic valve replacement, STS-PROM: Society of Thoracic Surgery-Predicted Risk of Mortality, NYHA: New York Heart Association, COPD: chronic occlusive pulmonary disease, CABG: coronary artery bypass grafting, PCI: percutaneous coronary intervention, DMU: Dokkyo Medical University
