# Supplementary material for: Contemporary short-term outcomes of surgery for aortic stenosis: transcatheter vs. surgical aortic valve replacement
Source: Gen Thorac Cardiovasc Surg. 2021 Jun 22;70(2):124–31. doi: 10.1007/s11748-021-01672-8 (PMC8817997; doi:10.1007/s11748-021-01672-8)
Supplement: Supplementary file 3 — Supplementary file3 (DOCX 18 KB) [file 11748_2021_1672_MOESM3_ESM.docx]

**Supplemental Table 3. Cause of death in the Low-risk Group**

Postoperative day Cause of death Group PVL ≥ 1 PVL ≥ 2

132 gastro-intestinal ischemia TAVR yes no

180 stroke TAVR yes no

219 heart failure TAVR yes yes

340 cancer TAVR yes no

385 heart failure, sepsis TAVR yes yes

414 pneumonia TAVR yes yes

425 unknown SAVR no no

684 unknown TAVR yes yes

TAVR: transcatheter aortic valve replacement, SAVR: surgical aortic valve replacement, PVL: paravalvular leakage
