# Supplementary material for: Contemporary short-term outcomes of surgery for aortic stenosis: transcatheter vs. surgical aortic valve replacement
Source: Gen Thorac Cardiovasc Surg. 2021 Jun 22;70(2):124–31. doi: 10.1007/s11748-021-01672-8 (PMC8817997; doi:10.1007/s11748-021-01672-8)
Supplement: Supplementary file 4 — Supplementary file4 (DOCX 25 KB) [file 11748_2021_1672_MOESM4_ESM.docx]

**Supplemental Table 4. Baseline Patient Characteristics (Low-risk Group) after Propensity Score Matching.**

**TAVR SAVR**

**Characteristics (N = 23) (N = 23) p value**

Age --- year 79.3 ± 3.6 79.0 ± 3.5 0.742

Female sex --- no. (%) 12 (52.2) 11 (47.8) 1.000

Body-mass index 22.2 ± 4.2 23.1 ± 3.3 0.458

STS-PROM 2.5 ± 0.5 2.7 ± 0.9 0.235

NYHA class III or IV --- no. (%) 4 (17.4) 2 ( 8.7) 0.665

Coronary artery disease --- no. (%) 2 ( 8.7) 4 (17.4) 0.665

Triple vessel disease and/or left main trunk disease --- no (%) 0 ( 0) 0 ( 0) -

Cerebral vascular disease / Carotid disease --- no. (%) 5 (21.7) 6 (26.1) 1.000

Peripheral vascular disease --- no. (%) 1 ( 4.3) 1 ( 4.3) 1.000

COPD --- no. (%) 3 (13.0) 4 (17.4) 1.000

creatinine > 2 mg/dl --- no. (%) 0 ( 0) 1 ( 4.3) 1.000

Hemodialysis --- no. (%) 0 ( 0) 0 ( 0) -

Diabetes --- no. (%) 8 (34.8) 6 (26.1) 0.749

Atrial fibrillation --- no (%) 1 ( 4.3) 4 (17.4) 0.346

Previous cardiovascular surgery --- no. (%) 0 ( 0) 0 ( 0) -

Bicuspid aortic valve --- no. (%) 1 ( 4.3) 0 ( 0) 1.000

Mitral insufficiency ≥ moderate --- no. (%) 1 ( 4.3) 0 ( 0) 1.000

Left ventricular ejection fraction --- % 60.1 ± 11.2 60.2 ± 8.1 0.992

Left ventricular ejection fraction < 30 ---no. (%) 1 ( 4.3) 0 ( 0) 1

Emergent / Urgent operation --- no. (%) 0 ( 0) 0 ( 0) -

Concomitant CABG / TAVR + PCI --- no. (%) 2 (8.7) 4 (17.4) 0.665

Institution: DMU --- no. (%) 15 (65.2) 9 (39.1) 0.139

TAVR: transcatheter aortic valve replacement, SAVR: surgical aortic valve replacement, STS-PROM: Society of Thoracic Surgery-Predicted Risk of Mortality, NYHA: New York Heart Association, COPD: chronic occlusive pulmonary disease, CABG: coronary artery bypass grafting, PCI: percutaneous coronary intervention, DMU: Dokkyo Medical University
