# Supplementary material for: Contemporary short-term outcomes of surgery for aortic stenosis: transcatheter vs. surgical aortic valve replacement
Source: Gen Thorac Cardiovasc Surg. 2021 Jun 22;70(2):124–31. doi: 10.1007/s11748-021-01672-8 (PMC8817997; doi:10.1007/s11748-021-01672-8)
Supplement: Supplementary file 5 — Supplementary file5 (PPTX 49 KB) [file 11748_2021_1672_MOESM5_ESM.pptx]

## Slide 1
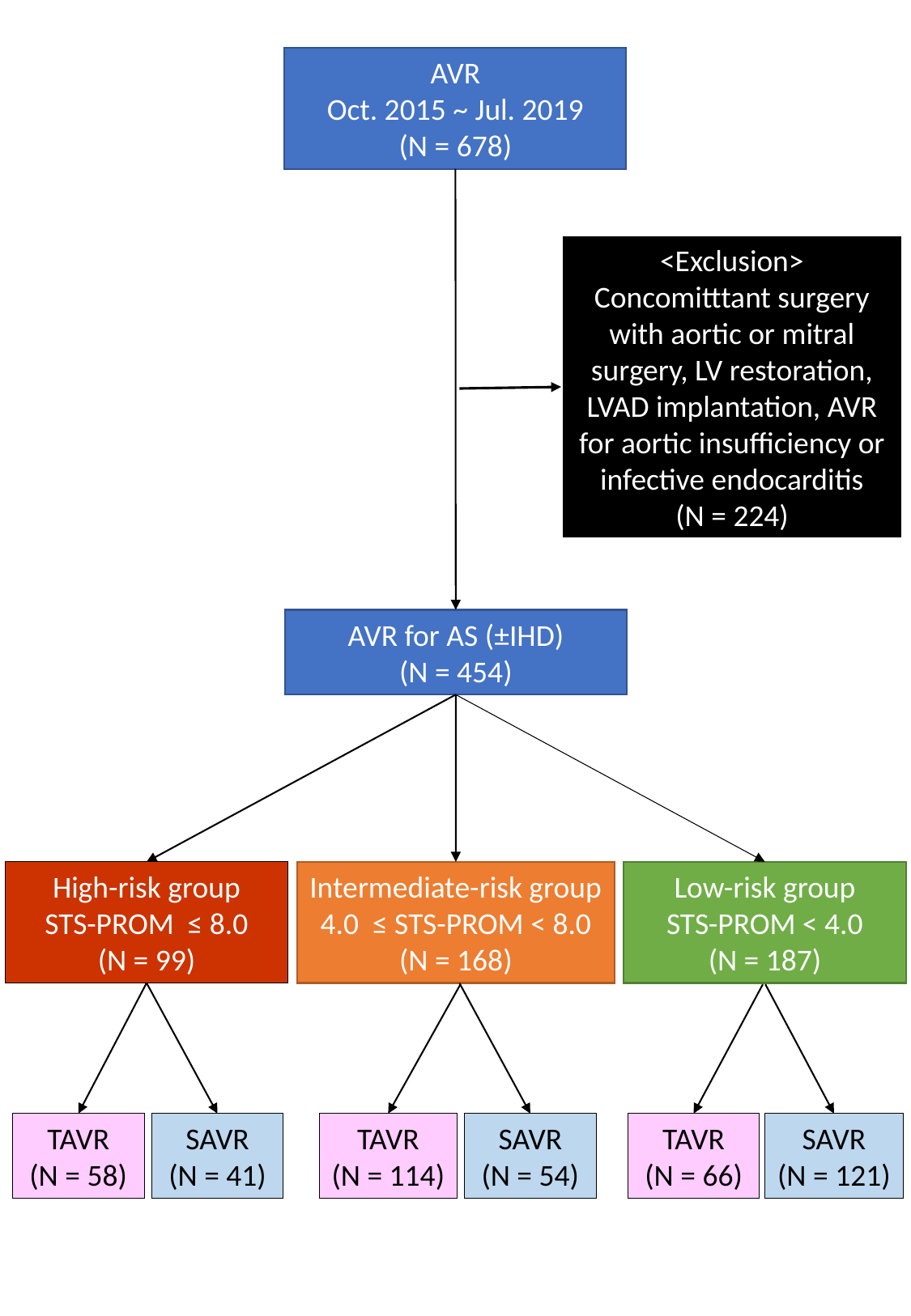

AVR
Oct. 2015 ~ Jul. 2019
(N = 678)
<Exclusion>
Concomitttant surgery with aortic or mitral surgery, LV restoration, LVAD implantation, AVR for aortic insufficiency or infective endocarditis
(N = 224)
AVR for AS (±IHD)
(N = 454)
High-risk group
STS-PROM ≤ 8.0
(N = 99)
Intermediate-risk group
4.0 ≤ STS-PROM < 8.0
(N = 168)
Low-risk group
STS-PROM < 4.0
(N = 187)
SAVR
(N = 41)
SAVR
(N = 54)
SAVR
(N = 121)
TAVR
(N = 58)
TAVR
(N = 114)
TAVR
(N = 66)
