# Supplementary material for: Contemporary short-term outcomes of surgery for aortic stenosis: transcatheter vs. surgical aortic valve replacement
Source: Gen Thorac Cardiovasc Surg. 2021 Jun 22;70(2):124–31. doi: 10.1007/s11748-021-01672-8 (PMC8817997; doi:10.1007/s11748-021-01672-8)
Supplement: Supplementary file 6 — Supplementary file6 (PPTX 70 KB) [file 11748_2021_1672_MOESM6_ESM.pptx]

## Slide 1
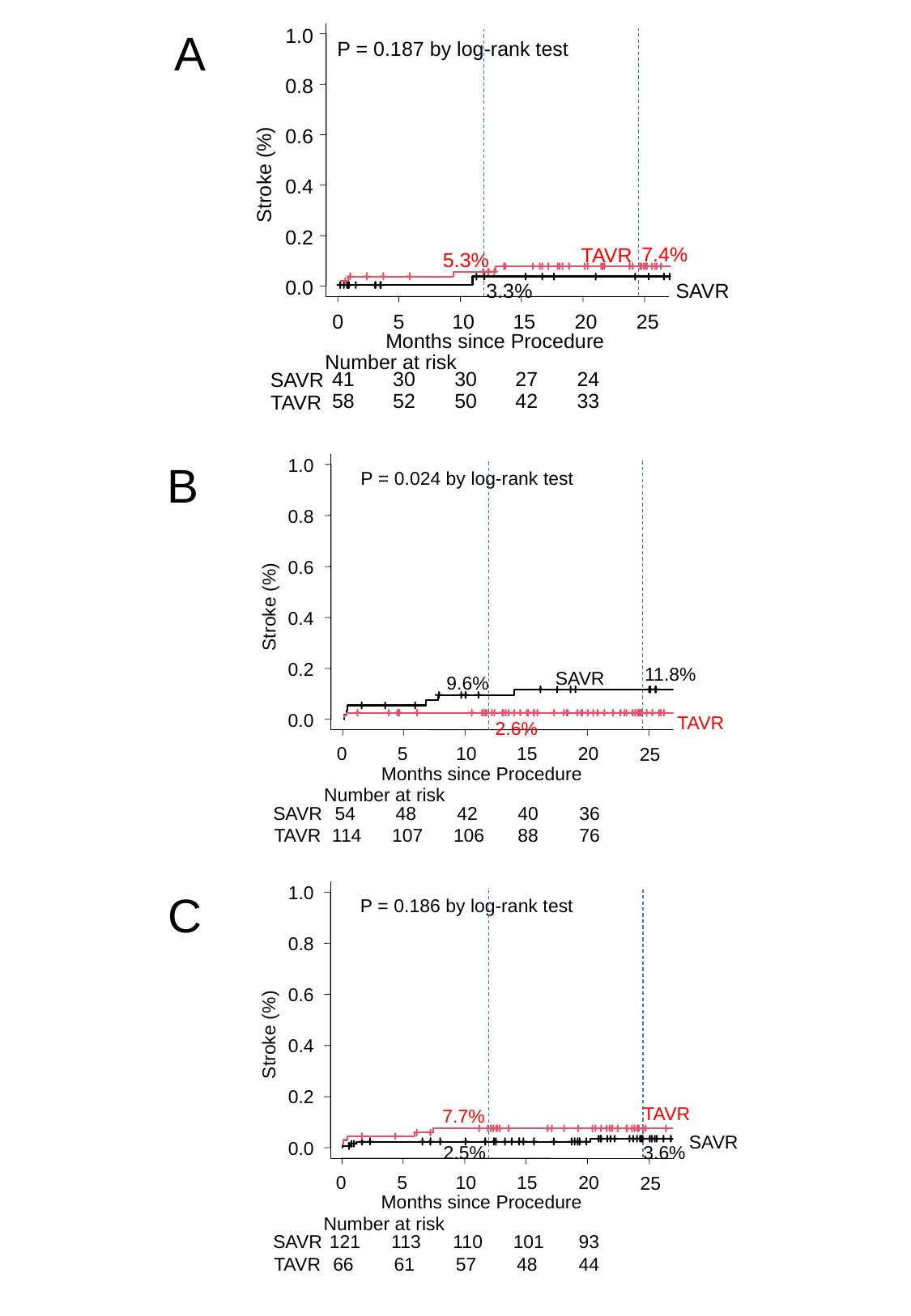

A
1.0
P = 0.187 by log-rank test
0.8
0.6
Stroke (%)
0.4
0.2
7.4%
TAVR
5.3%
0.0
3.3%
SAVR
0
5
10
15
20
25
Months since Procedure
Number at risk
41
30
30
27
24
SAVR
58
52
50
42
33
TAVR
B
1.0
P = 0.024 by log-rank test
0.8
0.6
Stroke (%)
0.4
0.2
11.8%
SAVR
9.6%
0.0
TAVR
2.6%
0
5
10
15
20
25
Months since Procedure
Number at risk
54
48
42
40
36
SAVR
TAVR
114
107
106
88
76
C
1.0
P = 0.186 by log-rank test
0.8
0.6
Stroke (%)
0.4
0.2
TAVR
7.7%
SAVR
0.0
2.5%
3.6%
0
5
10
15
20
25
Months since Procedure
Number at risk
121
113
110
101
93
SAVR
66
61
57
48
44
TAVR
