# Supplementary material for: Contemporary short-term outcomes of surgery for aortic stenosis: transcatheter vs. surgical aortic valve replacement
Source: Gen Thorac Cardiovasc Surg. 2021 Jun 22;70(2):124–31. doi: 10.1007/s11748-021-01672-8 (PMC8817997; doi:10.1007/s11748-021-01672-8)
Supplement: Supplementary file 7 — Supplementary file7 (PPTX 72 KB) [file 11748_2021_1672_MOESM7_ESM.pptx]

## Slide 1
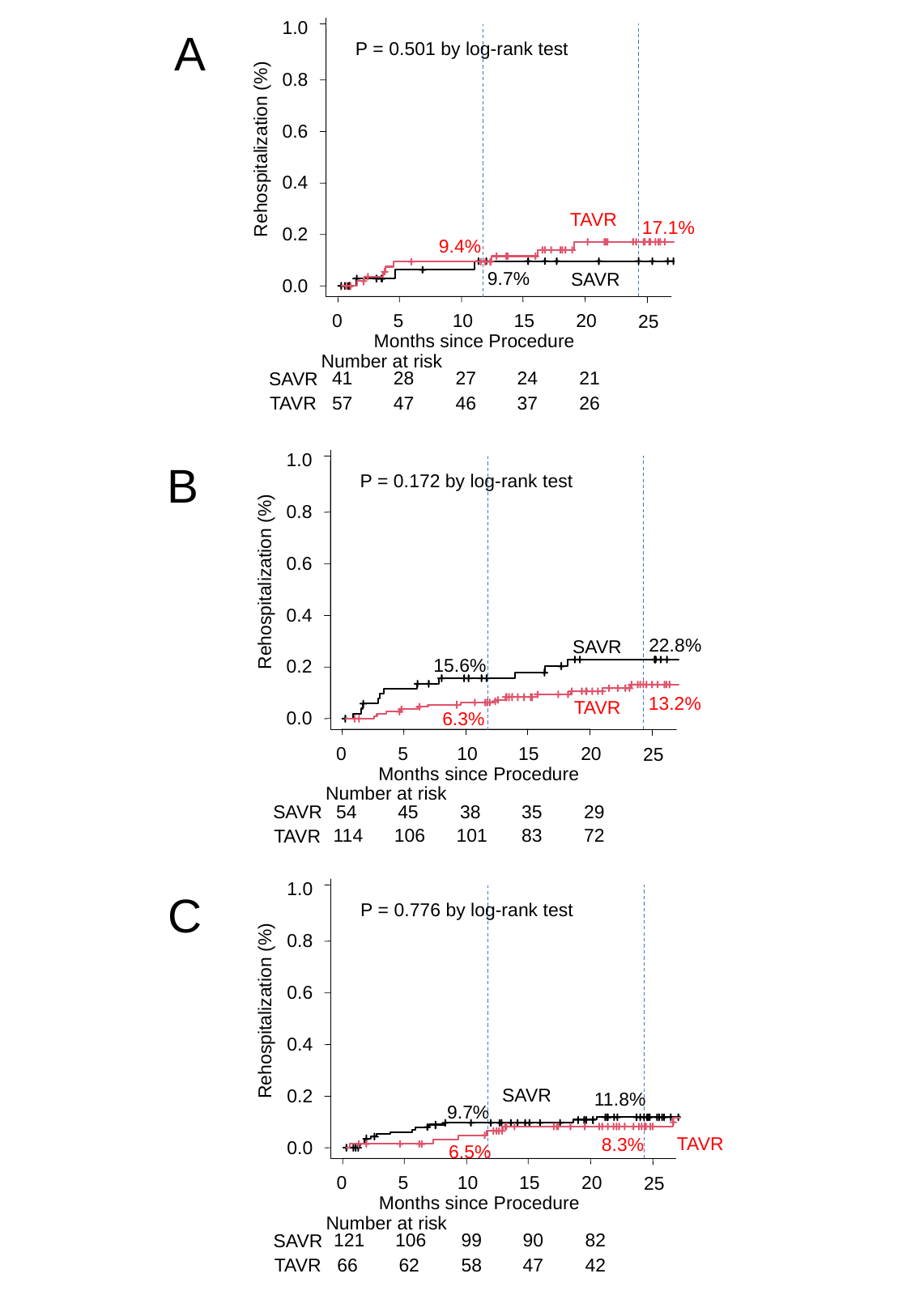

1.0
P = 0.501 by log-rank test
0.8
0.6
Rehospitalization (%)
0.4
TAVR
17.1%
0.2
9.4%
9.7%
SAVR
0.0
0
5
10
15
20
25
Months since Procedure
Number at risk
41
28
27
24
21
SAVR
57
47
46
37
26
TAVR
A
1.0
P = 0.172 by log-rank test
0.8
0.6
Rehospitalization (%)
0.4
22.8%
SAVR
15.6%
0.2
13.2%
TAVR
0.0
6.3%
0
5
10
15
20
25
Months since Procedure
Number at risk
SAVR
54
45
38
35
29
114
106
101
83
72
TAVR
B
1.0
P = 0.776 by log-rank test
0.8
0.6
Rehospitalization (%)
0.4
SAVR
0.2
11.8%
9.7%
TAVR
8.3%
0.0
6.5%
0
5
10
15
20
25
Months since Procedure
Number at risk
121
106
99
90
82
SAVR
TAVR
66
62
58
47
42
C
