# Supplementary material for: Contemporary short-term outcomes of surgery for aortic stenosis: transcatheter vs. surgical aortic valve replacement
Source: Gen Thorac Cardiovasc Surg. 2021 Jun 22;70(2):124–31. doi: 10.1007/s11748-021-01672-8 (PMC8817997; doi:10.1007/s11748-021-01672-8)
Supplement: Supplementary file 8 — Supplementary file8 (PPTX 70 KB) [file 11748_2021_1672_MOESM8_ESM.pptx]

## Slide 1
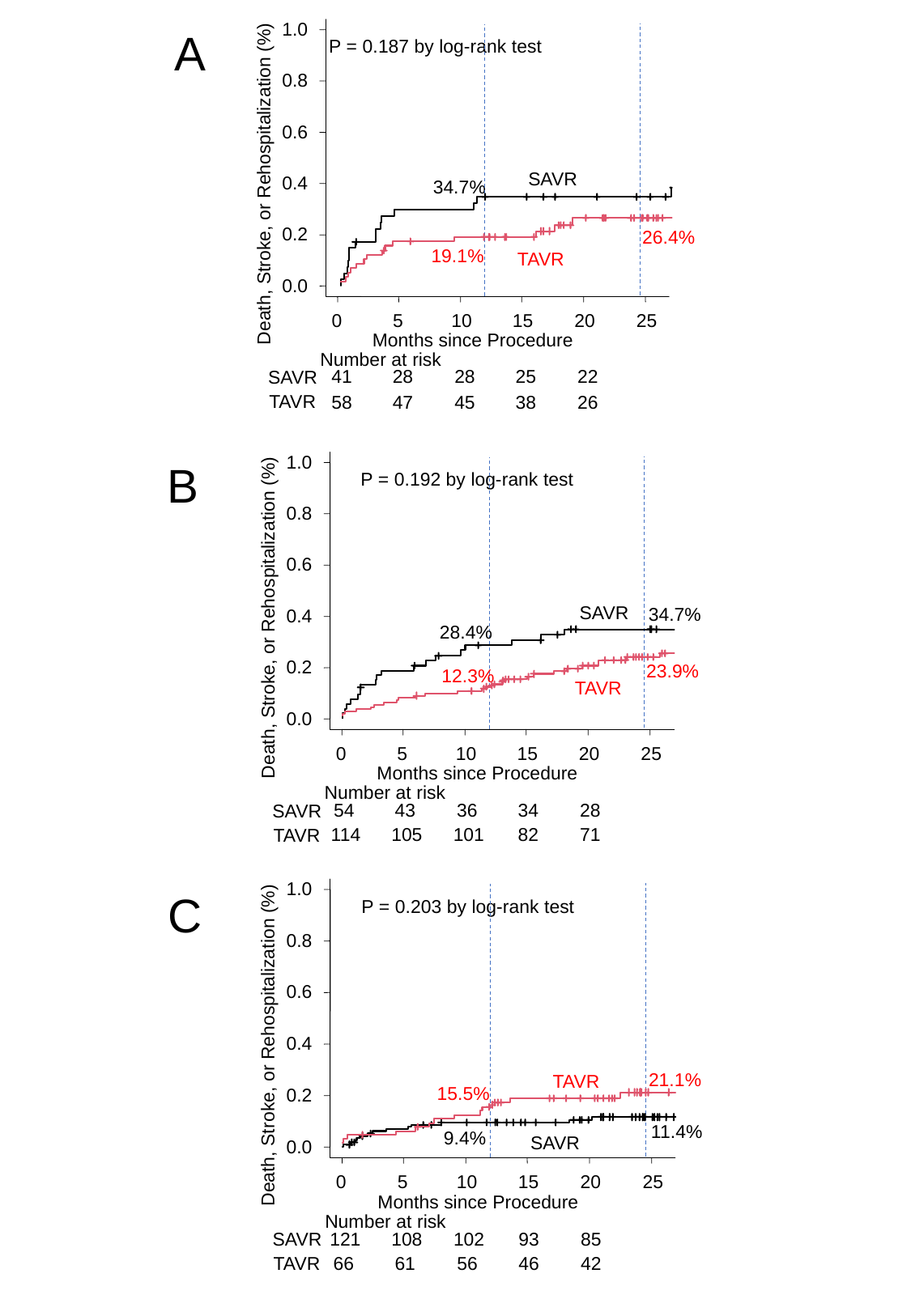

A
1.0
P = 0.187 by log-rank test
0.8
0.6
SAVR
0.4
Death, Stroke, or Rehospitalization (%)
34.7%
0.2
26.4%
19.1%
TAVR
0.0
0
5
10
15
20
25
Months since Procedure
Number at risk
41
28
28
25
22
SAVR
TAVR
58
47
45
38
26
B
1.0
P = 0.192 by log-rank test
0.8
0.6
SAVR
34.7%
0.4
Death, Stroke, or Rehospitalization (%)
28.4%
0.2
23.9%
12.3%
TAVR
0.0
0
5
10
15
20
25
Months since Procedure
Number at risk
54
43
36
34
28
SAVR
114
105
101
82
71
TAVR
1.0
P = 0.203 by log-rank test
0.8
0.6
0.4
Death, Stroke, or Rehospitalization (%)
21.1%
TAVR
15.5%
0.2
11.4%
9.4%
SAVR
0.0
0
5
10
15
20
25
Months since Procedure
Number at risk
121
108
102
93
85
SAVR
66
61
56
46
42
TAVR
C
