# Supplementary material for: Contemporary short-term outcomes of surgery for aortic stenosis: transcatheter vs. surgical aortic valve replacement
Source: Gen Thorac Cardiovasc Surg. 2021 Jun 22;70(2):124–31. doi: 10.1007/s11748-021-01672-8 (PMC8817997; doi:10.1007/s11748-021-01672-8)
Supplement: Supplementary file 9 — Supplementary file9 (PPTX 41 KB) [file 11748_2021_1672_MOESM9_ESM.pptx]

## Slide 1
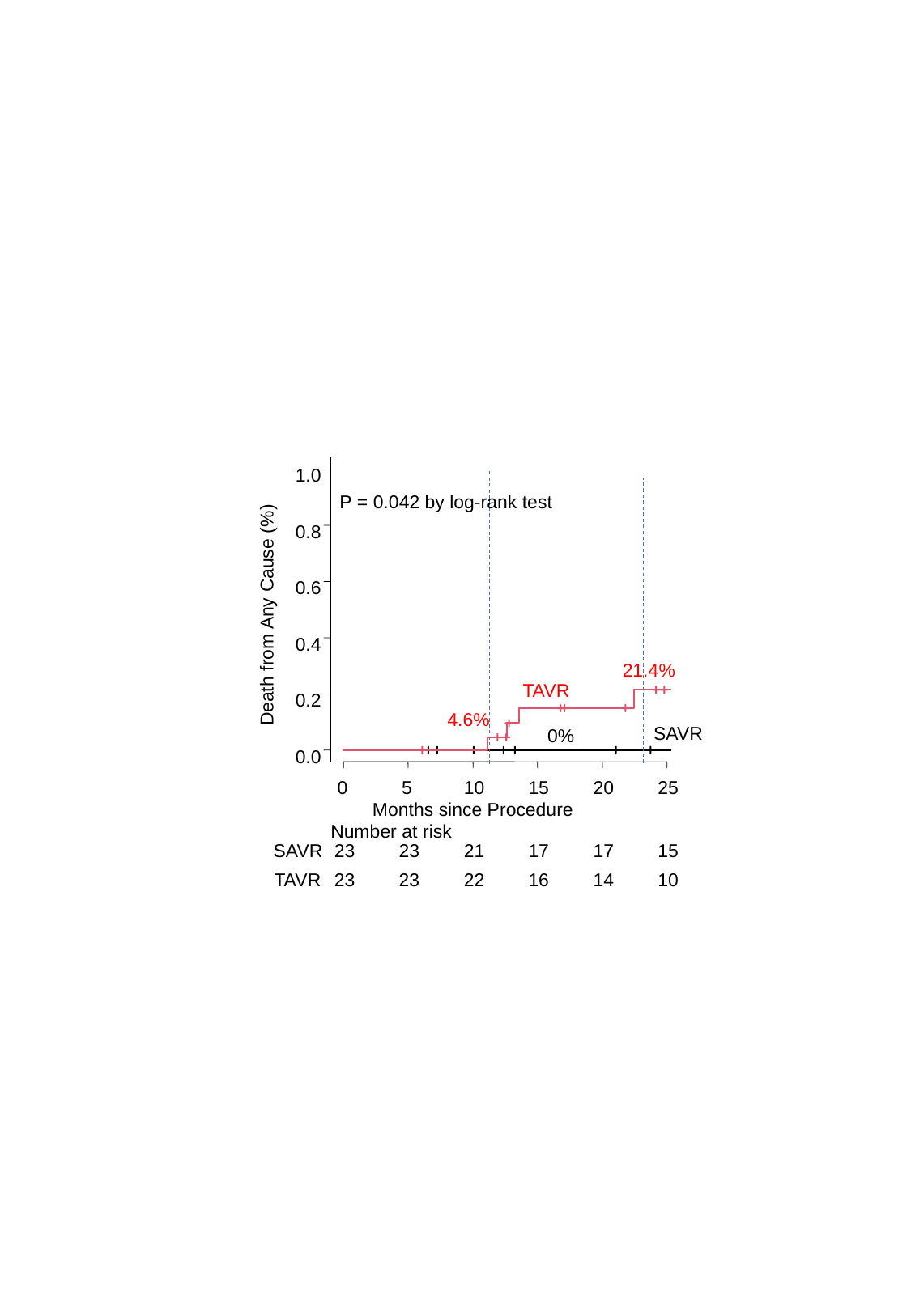

1.0
P = 0.042 by log-rank test
0.8
0.6
Death from Any Cause (%)
0.4
21.4%
TAVR
0.2
4.6%
SAVR
0%
0.0
0
5
10
15
20
25
Months since Procedure
Number at risk
SAVR
23
23
21
17
17
15
TAVR
23
23
22
16
14
10
