# Supplementary material for: Contemporary short-term outcomes of surgery for aortic stenosis: transcatheter vs. surgical aortic valve replacement
Source: Gen Thorac Cardiovasc Surg. 2021 Jun 22;70(2):124–31. doi: 10.1007/s11748-021-01672-8 (PMC8817997; doi:10.1007/s11748-021-01672-8)
Supplement: Supplementary file 10 — Supplementary file10 (DOCX 14 KB) [file 11748_2021_1672_MOESM10_ESM.docx]

**Supplemental Figure Legends**

**Supplemental Figure 1** Study design. AVR: aortic valve replacement, IHD: ischemic heart disease, TAVR: transcatheter AVR, SAVR: surgical AVR, STS-PROM: Society of Thoracic Surgery-Predicted Risk of Mortality, LV: left ventricle, LVAD: left ventricular assist device

**Supplemental Figure 2** Stroke rate in (A) high-risk group, (B) intermediate-risk group, and (C) low-risk group

**Supplemental Figure 3** Rehospitalization rate in (A) high-risk group, (B) intermediate-risk group, and (C) low-risk group

**Supplemental Figure 4** The combined outcome of death, stroke or rehospitalization in (A) high-risk group, (B) intermediate-risk group, and (C) low-risk group

**Supplemental Figure 5** All-cause mortality in low-risk group after the propensity score matching
